# Fangji Dihuang formulation ameliorated DNCB-induced atopic dermatitis-like skin lesions by IL-17 signaling pathway: integrating network analysis and experimental validation

**DOI:** 10.3389/fphar.2023.1220945

**Published:** 2023-11-27

**Authors:** Wenting Zhao, Honghong Jiang, Yunfan Gu, Weiming Zhang, Shijie Bao, Ming Dai, Bilin Dong, Ya Yang, Ke Li, Li Qin, Xianyu Zeng

**Affiliations:** ^1^ Department of Dermatology, Traditional Chinese and Western Medicine Hospital of Wuhan, Tongji Medical college, Huazhong University of Science and Technology, Wuhan, Hubei, China; ^2^ The First Clinical College, Hubei University of Chinese Medicine, Wuhan, China; ^3^ Department of Dermatology, The Second People’s Hospital of Guiyang, Guizhou, China

**Keywords:** Fangji Dihuang formulation, atopic dermatitis, inflammation, single-cell RNA-seq, network analysis, RNA sequence

## Abstract

**Background:** The Fangji Dihuang formulation (FJDHF) is a widely recognized Traditional Chinese Medicine (TCM) formula that consists of five plant drugs: Stephaniae Tetrandrae Radix, Cinnamomi Ramulus, Rehmanniae Radix, Saposhnikoviae Radix, and Glycyrrhiza Urensis Fisch. This formulation has been known to exhibit clinical therapeutic effects in the treatment of inflammatory skin diseases. However, there is a lack of pharmacological research on its anti-atopic dermatitis (AD) activity.

**Methods:** To investigate the potential anti-AD activity of FJDHF, DNCB was used to induce AD-like skin inflammation in the back of mice. Following successful modeling, the mice were administered FJDHF orally. The extent of the inflammatory skin lesions was recorded at day 4, 7, 14 and 28. UHPLC-Q-Exactive Orbitrap MS was used to identify and match the compounds present in FJDHF with ITCM, TCMIP and TCMSID. In silico predictions of potential target proteins of the identified compounds were obtained from SwishTargetPrediction, ITCM and TargetNet databases. AD-related genes were identified from GSE32924 data set, and FJDHF anti-AD hub genes were identified by MCODE algorithm. ClueGo enrichment analysis was employed to identify the core pathway of FJDHF’s anti-AD effect. To further investigate the anti-AD effect of FJDHF, single-cell RNA sequencing data set (GSE148196) from AD patients was analyzed to determine the target cells and signaling pathways of FJDHF in AD. Finally, rt-PCR, flow cytometry, and mouse back skin RNA sequencing were utilized to validate our findings.

**Results:** FJDHF was found to be effective in improving the degree of the AD-like lesions in the mice. Network pharmacological analysis revealed the core pathway of FJDHF to be the IL-17 signaling pathway, which is interactively associated with cytokines. Single-cell RNA sequencing analysis suggested that FJDHF may play an anti-AD role by influencing dendritic cells. Flow cytometry and rt-PCR results showed that FJDHF can reduce the influence of AD sample of IL-4, IFN-γ and the expression of IL-17. The RNA sequencing of mouse back skin also confirmed our conclusion.

**Conclusion:** FJDHF may inhibit DNCB-induced AD-like skin inflammation in mice by inhibiting the IL-17 signaling pathway. Thus, FJDHF can be considered as a potential therapeutic agent for AD.

## 1 Introduction

Atopic dermatitis (AD) is a highly recurrent, chronic inflammatory skin disease and is the most common non-fatal skin condition leading to substantial patient burden (Ständer, 2021). While incidence appears to have plateaued in developed countries, the prevalence of AD is on the rise in developing countries (Yi et al., 2019). Its prevalence is estimated to be between 15%–20% in children and up to 10% in adults (Laughter et al., 2021). Some of the challenges in AD management are universal, and, unfortunately, there is no cure yet. Biologics and oral small-molecule JAK1 inhibitors largely control the symptoms of AD but imply high costs in resource-limited areas (Kuznik et al., 2017; Cheng and Silverberg, 2019; Ratchataswan et al., 2021). The average annual cost for a child with AD in Asia is estimated to be US $7,943, including healthcare expenditures and informal care costs (Olsson et al., 2020), adding to the economic burden for children and their families. The average direct cost of AD was $4,411 in adults and adolescents, while the average indirect cost was $9,068 per year (Fasseeh et al., 2022). Furthermore, the severity of AD also has a corresponding impact on work efficiency; moderate AD patients lose an average of 9.6 h per week, while severe AD patients lose 19.0 h (Andersen et al., 2020). Another main medication for the treatment of AD is glucocorticoids. In many patients with mild to moderate AD, significant adverse reactions of glucocorticoid lead to corticosteroid phobia, which results in insufficient or no prescription of topical corticosteroids (TCS) (Li et al., 2018). Systemic corticosteroids use also implies more adverse reactions and rebound flaring of disease (Brar et al., 2019). As a result, traditional medicine continues to play an important role in meeting healthcare needs in many developing countries (Yi et al., 2019).

Chinese herbal therapy, as one of the complementary and alternative therapies, has a well-developed theoretical basis. According to several systematic reviews, Chinese herbal therapy can improve the size and severity of skin lesions, as well as sleep quality in AD patients (Cai et al., 2022; Li et al., 2022). Fangji Dihuang formulation (FJDHF), recorded in the ‘Synopsis of Golden Chamber’, is composed of five botanical drugs: *Stephaniae Tetrandrae Radix, Cinnamomi Ramulus*, *Rehmanniae Radix, Saposhnikoviae Radix*, and *Glycyrrhiza uralensis Fisch.* Its efficacy has been widely recognized by both doctors and patients, likely due to the anti-inflammatory, anti-allergic, and antioxidant effects of these botanical drugs and their active ingredients (Liu et al., 2022; Lu et al., 2022). Despite the lack of knowledge on the pharmacological studies and potential mechanisms of anti-AD in Fangji Dihuang formulation (FJDHF), this study integrated network pharmacology and bioinformatics analysis to identify the target and cell of action of FJDHF, which was then verified through animal experiments.

## 2 Methods

**FIGURE 1:**
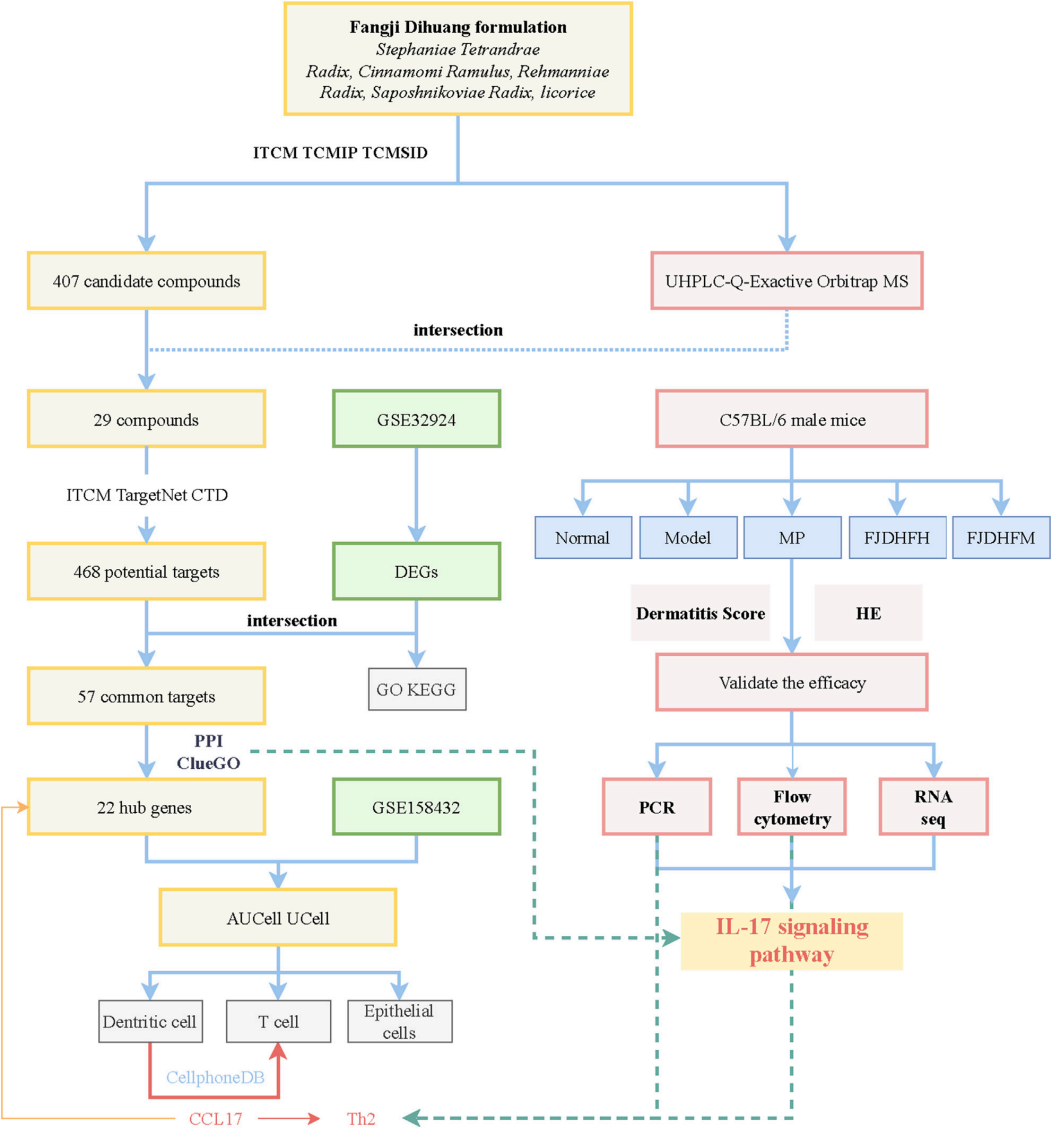
Workflow figure of this study.

The flow of this work is plotted in Figure 1.

### 2.1 Preparation of FJDHF

In our experiments, the preparations of FJDHF were provided by the Department of Pharmacy of Wuhan No.1 Hospital. These preparations were consisted of five ingredients: 10 g dried roots of *Stephania tetrandra* S.Moore [Menispermaceae], 10 g dried twigs of *Cinnamomum cassia* Presl [Lauraceae], 10 g dried roots of *Saposhnikovia divaricata* (Turcz.) Schischk [Umbelliferae], 6 g dried roots of *Glycyrrhiza uralensis* Fisch [Fabaceae Lindl], along with 60 g fresh tuberous root of *Rehmannia glutinosa* Libosch [Scrophulariales]. The mass ratio is 5: 5: 5: 3: 30. These ingredients were authenticated by Professor Xu Hongfeng from the Department of Pharmacy of Traditional Chinese and Western Medicine Hospital of Wuhan.

The FJDHF extraction process is as follows: a total of 96 g of the five ingredients is taken and soaked in twice the amount of water for 0.5 h. Then, 10 times the amount of water is added, and the mixture is decocted twice, with each decoction lasting 1 h. The resulting decoctions are filtered and combined. The filtrate is then concentrated under reduced pressure to achieve a relative density of about 1.02–1.05 (40 °C). To dissolve the concentrate, 5 mL of water is added and boiled. This solution is then added to the concentrated solution, followed by the addition of water up to 100 mL. The mixture is filtered to obtain an initial extraction solution of 50 mL. Finally, the solution is dried using a rotary evaporator, resulting in 27.4 g (with 96 g raw ingredients) of freeze-dried powder of the FJDHF formulation.

Based on clinical experience and references from “Synopsis of the Golden Chamber” the recommended dose of FJDHF freeze-dried powder for adults weighing 60 Kg is 27.4 g/d (with a raw ingredients dose of 96 g/d), which translates to 0.457 g/kg/d. To ensure a comparable drug dose ratio between humans and animals, we utilized the body surface area conversion formula used by Wei et al. This led us to determine a dosage of 4.1587 mg/g/d for mice, calculated as 457 mg * 70 kg * 0.0026/20 g (Wei et al., 2010). In our *in vivo* experiment, the FJDHF medium dose group (FJDHF-M) was administered a dose of 4.158 mg/kg/d, while the high-dose group (FJDHF-H) received a dose 1.5 times higher, amounting to 6.238 mg/kg/day.

To ensure compliance with pharmacopoeia standards and minimize batch variations, we employed the UHPLC-Q-Exactive Orbitrap MS method to analyze the components of the FJDHF freeze-dried powder. The operating method for UHPLC-Q-Exactive Orbitrap MS is as follows: After thawing the FJDHF-H solution on ice, it was vortexed for 30 s. The sample was then centrifuged at 4°C and 12,000 rpm for 15 min to obtain the supernatant. 300 μL of this supernatant was taken and mixed with 1000 μL of extraction solution (methanol: water in a 4:1 ratio), after which it was vortexed for 30 s and ultrasonicated in an ice water bath for 5 min. The sample was then left to stand at −40°C for 1 h before being centrifuged at 12,000 rpm at 4°C for 15 min. The supernatant was carefully taken and filtered using a 0.22 μm microporous filter membrane (Vanquish, Thermo Fisher Scientific, United States). Analysis was then conducted using an UPLC BEH C18 column (1.7 μm*2.1*100 mm) purchased from Waters, with an injection volume of 5 μL.

### 2.2 Mice

**FIGURE 2:**
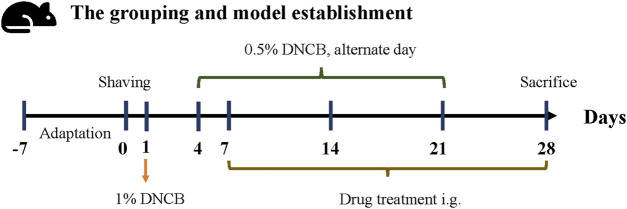
Modeling the methodology and group administration scheme of AD-like mice.

C57BL/6 male mice (Beijing Vital River Laboratory Animal Technology Co., Ltd.,China) aged 6–8 weeks were divided into 5 groups (n = 8) after adaptive feeding for 1 week: model group (Model), methylprednone control group (MP), FJDHF high-dose group (FJDHF-H), FJDHF medium-dose group (FJDHF-M), and normal control group. 2, 4-dinitrochlorobenzene (DNCB, Sigma, United States) was dissolved in acetone and olive oil (volume 3:1) before use. On day 0, each mouse was shaved on the back within a 2*2 cm area. On day 1, the back skin of all mice, except the normal control group (NC), were sensitized with 200 μL of a 1% DNCB solution. Starting from day 4, the back skin of all mice, except the normal control group, were repeatedly stimulated with 100 μL of a 0.5% DNCB solution for the duration of the experiment (days 4, 7, 9, 11, 13, 15, 17, 19 and 21) (Lee et al., 2020). After the seventh day, the FJDHF-H and FJDHF-M group received different concentrations of FJDHF by oral tube feeding daily, while the MP group was administered 10 mg/kg of methylprednone (CAS: 83-43-2, MCE, China) orally until the end of the experiment (day 28). The NC group did not receive any treatment. The specific modeling process and intervention methods are depicted in Figure 2. All mice were kept in a temperature-controlled (20°C–22°C) and humidity-controlled (55%) SPF environment, with free access to food and water. The animal experiment was ethically approved by the Animal Experiment Center of Hubei University of Traditional Chinese Medicine (HUCMS202108009).

### 2.3 Evaluation of dermatitis scores

The dermatitis index score of each mouse was assessed every 7 days starting on the seventh day, according to a modified version of the Eczema Area and Severity Index (EASI) (Chopra et al., 2017). The EASI score consists of five aspects: surface area, Erythema, Thickness, Excoriation, and Lichenification. Since the modeling area was nearly the same in each experimental group, we evaluated and scored only four items: Erythema, Thickness, Excoriation, and Lichenification. The severity of these symptoms was scored on a scale from 0 (mildest) to 3 (most severe) (Chen et al., 2021).

On days 4, 7, 14, and 28 of the experiment, photos of the mice’s back skin were taken simultaneously. Dermatologists who were not involved in the experiment then scored the symptoms based on the photos.

### 2.4 HE staining

On the 28th day, the mice were sacrificed, and the dorsal skin tissue was harvested and fixed in 4% paraformaldehyde for 24 h. The tissue was then dehydrated and embedded in paraffin before being cut into 5 μm-thick slices and stained with hematoxylin and eosin (H&E). The specific procedure involves soaking the slices in hematoxylin dye for 4 min after the dewaxing and hydration steps. Finally, the slices are dyed with eosin. The entire process is performed using a fully automatic staining machine (HistoCore SPECTRA ST, Leica, Germany).

### 2.5 Rt-PCR

According to the manufacturer’s instructions, total RNA was extracted from skin tissue samples using the MiniBEST Universal RNA Extraction Kit (Code# 9767, Takara, China) after grinding with liquid nitrogen. cDNA was synthesized using approximately 1 μg of RNA (Code# RR047A, Takara, China). SYBR Green (Code# RR820A Takara, China) was employed to determine the expression levels of IL-17, IL-4, and IFN-γ with GAPDH as endogenous controls, using a PCR system Step one Plus (Thermo Fisher, United States). The primer sequence is shown in Table 1. The reaction is carried out under the following conditions: Stage 1 predenaturation at 95°C for 30 s, followed by Stage 2 PCR reaction with 40 cycles at 95°C for 5 s and 60°C for 30 s. GAPDH was used as the internal control. The 2^-ΔΔCt method was used to calculate the relative expression of these mRNA.

**TABLE 1:** RT-PCR primers.

Gene	Primers sequence (5'- 3′)
IL17A-F	CAG​ACT​ACC​TCA​ACC​GTT​CCA
IL17A-R	ACA​ATC​GAG​GCC​ACG​CAG​GTG​CAG​C
IL4-F	GGT​CTC​AAC​CCC​CAG​CTA​GT
IL4-R	GCC​GAT​GAT​CTC​TCT​CAA​GTG​AT
IFNγ-F	CAG​GCC​ATC​AGC​AAC​AAC​ATA​AGC
IFNγ-R	AGC​TGG​TGG​ACC​ACT​CGG​ATG
GAPDH-F	AGG​TCG​GTG​TGA​ACG​GAT​TTG
GAPDH-R	GGG​GTC​GTT​GAT​GGC​AAC​A

### 2.6 Network analysis and bioinformatics analysis

#### 2.6.1 Prediction of putative FJDHF prescription targets

After downloading a total of ingredients with DrugLike Score greater than 0.5 from the ITCM website (Tian et al., 2023), Chemical Component from TCMIP (Xu et al., 2019) and TCMSID (Zhang et al., 2022), they were intersected with the ingredients identified by UHPLC-Q-Exactive Orbitrap MS. Four databases were consulted to identify known therapeutic targets for AD: the Swisstargetprediction database (Daina et al., 2019), the ITCM database, the TargetNet Database (Yao et al., 2016), and the Comparative Toxicogenomics Database (Davis et al., 2021).

#### 2.6.2 Data source

We obtained gene expression data (GSE32924) from the Gene Expression Omnibus (GEO) database to analyze the characteristic genes of atopic dermatitis (AD) in the skin lesions of AD patients and normal healthy people (Suárez-Fariñas et al., 2011). Additionally, single-cell RNA sequencing data (GSE158432) from 10 AD patients were downloaded and used to analyze the mechanism of FJDHF action (Bangert et al., 2021).

#### 2.6.3 Construct protein-protein interaction (PPI) network

The targets of FJDHF were compared to characteristic genes of AD, producing a Venn diagram for the common targets. To assess the priority of these common targets, they were imported into the String database (Szklarczyk et al., 2019) for network topology analysis. The resulting PPI network was visualized using Cytoscape software (Shannon et al., 2003). The Molecular Complex Detection (MCODE) algorithm was employed to identify highly connected network components, which were regarded as hub genes of FJDHF anti-AD.

#### 2.6.4 Molecular docking

We use the CB Dock2 online tool for molecular docking (Liu et al., 2022). The CCL17 protein structure can be downloaded from PubChem, and the MOL2 structure files for plant medicinal ingredients can be downloaded from the TCMSP website or PubChem.

#### 2.6.5 Functional enrichment analysis

The ClusterProfiler package was used to assess the GO (The Gene Ontology Consortium, 2019) and KEGG (Kanehisa et al., 2017) for DEGs in GSE32924, considering a |log2FC|≥1, False Discovery Rate (FDR) of <0.05 and *p*-values <0.01 as statistically significant. Furthermore, an analysis of hub genes enrichment was conducted using ClueGo plug-in on Cytoscape 3.6.2 (Bindea et al., 2009).

#### 2.6.6 Receiver operating characteristic (ROC) curve

The pROC package was used to plot receiver operating characteristic (ROC) curves and the selected core genes were evaluated by calculating the area under the ROC curve.

#### 2.6.7 Single cell RNA sequencing data set analysis

We downloaded single-cell RNA sequencing data (GSE158432) from the tissue of patients with AD. The Seurat (version 4.0) R software package was used for pretreatment, and the ScaleData function was employed to filter all genes. Subsequently, 30 principal components with significant statistical significance were selected as inputs of t-Distributed Neighbor Embedding (t-SNE). The PanglaoDB database was used to retrieve the immune cell marker genes, and the corresponding genes of various groups were intersected to identify the class groups of immune cells (Franzén et al., 2019). Finally, we employed the AUCell and UCell modules of the R package to evaluate the action sites of FJDHF. We then utilized CellPhoneDB to analyze and explore the relationship between action sites and other skin tissue cells. CellPhoneDB is a repository that breaks down ligand-receptor interactions between two cell types. During the analysis, we obtained a *p*-value (with a 10% threshold) for the cell-type-specific likelihood of the receptor-ligand complex based on the default settings of CellPhoneDB (Davidson et al., 2020; Kim et al., 2020).

### 2.7 Flow cytometry

Mice were dissected on a super-clean table, and the spleen was ground in a 70 μm strainer to obtain cell suspensions. For cell surface protein detection, cells were re-suspended in 100 μL cold DMEM medium with 10% FBS and 2% antibiotics, stimulated with leucocyte activators (including Brefeldin A, CAT#550583, BD, United States) for 4–6 h, and 0.5 μL Fixable Viability Stain 620 (CAT# 564996, BD, United States) was added and incubated at 4°C for 20 min. Subsequently, 1 mL PBS and 250 g (gravity acceleration) were added for centrifugation to remove supernatant. After re-suspension, 2 μL FC segment receptor blocker (CAT# 553141, BD, United States) was added and incubated for 15 min at 4°C in the dark. Cells were then incubated with CD3 and CD4 fluorescent dye conjugated antibodies (CAT# 561798,553051, BD, United States) in the dark on ice for 30 min. After washing two times with 1×PBS and 0.1%BSA, the cells were suspended in 100 μL washing buffer. For intracellular protein detection, the proposed process using the Fixation/Permeabilization kit (CAT# 554714, BD, United States) was followed, and IL-4, IL-17 and IFN-γ fluorescent coupling antibodies (CAT# 564007557649, BD, United States) were then incubated for 40 min. After washing twice, the cells were re-suspended in 100 μL 1×PBS and 0.1%BSA for flow cytometry analysis (Beckman Coulter, United States). FlowJo software was used to analyze the data.

### 2.8 RNA sequencing of skin in dorsal skin and enrichment analysis

Total mouse dorsal skin samples were subjected to RNA isolation and purification via TRIzol (CAT#15596018, ThermoFisher, United States). The quantity and quality of the total RNA were then assessed using NanoDrop ND-1000 (NanoDrop, Wilmington, DE, United States) and RNA integrity was evaluated using the Bioanalyzer 2100 (Agilent, CA, United States). Dynabeads Oligo (dT) Beads (cat.25-61005, Thermo Fisher, United States) were then employed in two rounds of purification for mRNA capture containing polyadenylated (PolyA) sequences. The captured mRNA was fragmented under high temperature utilizing the NEBNextR Magnesium RNA Fragmentation Module (CAT#E6150S, United States). cDNA synthesis was performed on the fragmented RNA via Invitrogen SuperScriptTM II Reverse Transcriptase (CAT#1896649, CA, United States). *E. coli* DNA Polymerase I (NEB, CAT#m0209, United States) and RNase H (CAT#m0297, NEB, United States) were utilized for two-strand synthesis in order to transform the double strands of DNA and RNA into DNA double strands. Concurrently, dUTP Solution (CAT#R0133, Thermo Fisher, United States) was added to the two-stranded DNA and the ends were completed to flat ends through the addition of an adenosine base to each end, allowing terminal junction with a thymine base. Magnetic beads were then employed to screen and purify fragment size. The two chains were digested by UDG enzyme (CAT#m0280, NEB, United States) and pre-denatured through PCR at 95°C for 3 min, followed by denaturation at 98°C for a total of 8 cycles of 15 s each, annealing at 60°C for 15 s, extension at 72°C for 30 s, which were then finally extended at 72°C for 5 min. This resulted in the formation of a fragment size library of 300bp±50bp (chain-specific library). Subsequently, an Illumina NovaseqTM 6000 was used to perform double-end sequencing in PE150 mode according to the relevant protocols. In this project, fold change ≥2 and q value < 0.05 are used as the threshold criteria for screening differentially expressed genes. Enrichment analysis uses the scheme in 2.7.4.

### 2.9 Statistical analysis

The data are represented as the mean standard measurement error (SEM). One-way analysis of variance was used for comparisons among multiple groups and a *t*-test was used for paired comparisons within groups. All statistical analyses were performed using GraphPad Prism 8.0 software (GraphPad Software, United States) and R processing. *p* < 0.05 was considered to be statistically significant.

## 3 Results

### 3.1 FJDHF attenuated the dermatitis score and reduced epidermal thickness in AD-like mice

On day 4, the group treated with DNCB exhibited various skin reactions, including epidermal thickening, exfoliation, exudation, and application site bleeding. The scores for these reactions were significantly higher than those of the NC group (4.37 ± 0.74 vs. 0.50 ± 0.53, *p* < 0.05). However, there were no statistically significant differences between the other groups and the Model group, except the NC group (*p* > 0.05).

**FIGURE 3:**
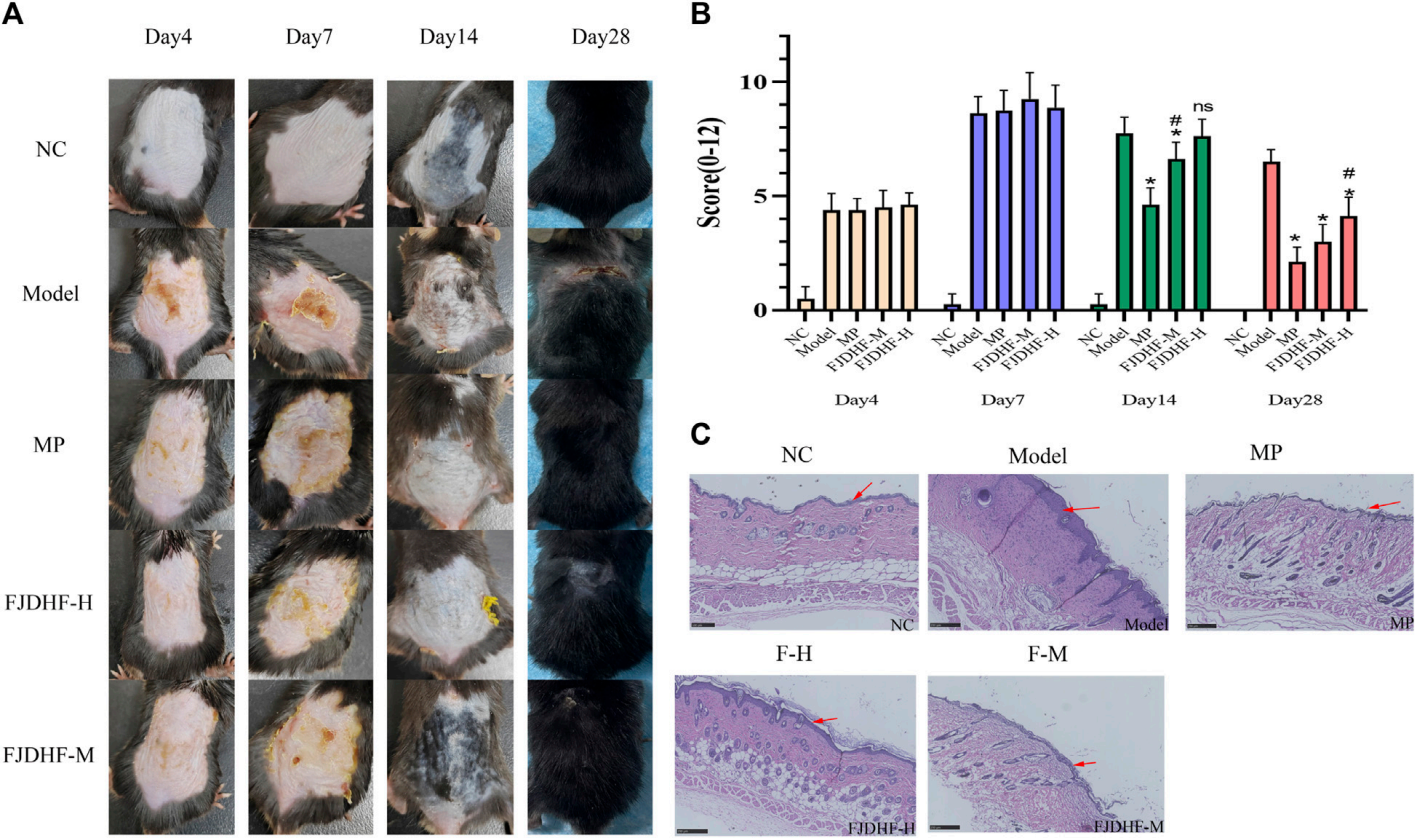
Fangji Dihuang formulation improved skin lesions in DNCB-induced AD mice. **(A)** On the Day 4, Day 7, Day 14, and Day 28, photographs were taken of the back skin of each group; **(B)** Dermatitis scores for each group, NC: normal control; Model: DNCB sensitized mice; MP: methylprednone (10 mg/kg) + DNCB; FJDHF-H: FJDHF-High Dose; FJDHF-M: FJDHF-Middle Dose. * Means vs Model group, *p* < 0.05, # means vs MP group, *p* < 0.05; ns Means vs Model group, *p* > 0.05 **(C)** HE staining of the back skin of mice in each group.

Furthermore, starting from day 14, both the MP group (4.62 ± 0.73) and the FJDHF-M group (6.62 ± 0.74) demonstrated significant reductions in scores compared to the Model group (7.75 ± 0.70) (*p* < 0.05). The degree of reduction in the MP group was superior to that of the FJDHF group (*p* < 0.05). On day 21, the lesions were similar to those on day 14. On the 28th day, the FJDHF-M group (3.00 ± 0.76) exhibited a significant difference compared to the Model group (6.50 ± 0.53) (*p* < 0.05). However, it is worth noting that the MP group had a more pronounced effect in relieving skin lesions compared to the FJDHF group (*p* < 0.05) (Figures 3A, B).

### 3.2 FJDHF can reduce epidermal thickening in DNCB induced atopic dermatitis model mice

The H&E staining sections of the back skin of each group were observed under an optical microscope. As demonstrated in Figure 3C DNCB significantly increased the thickness of the epidermis in mice compared to the NC group. Additionally, both FJDHF and MP were shown to significantly improve epidermal thickening and inflammatory cell infiltration.

### 3.3 Target genes of FJDHF

**FIGURE 4:**
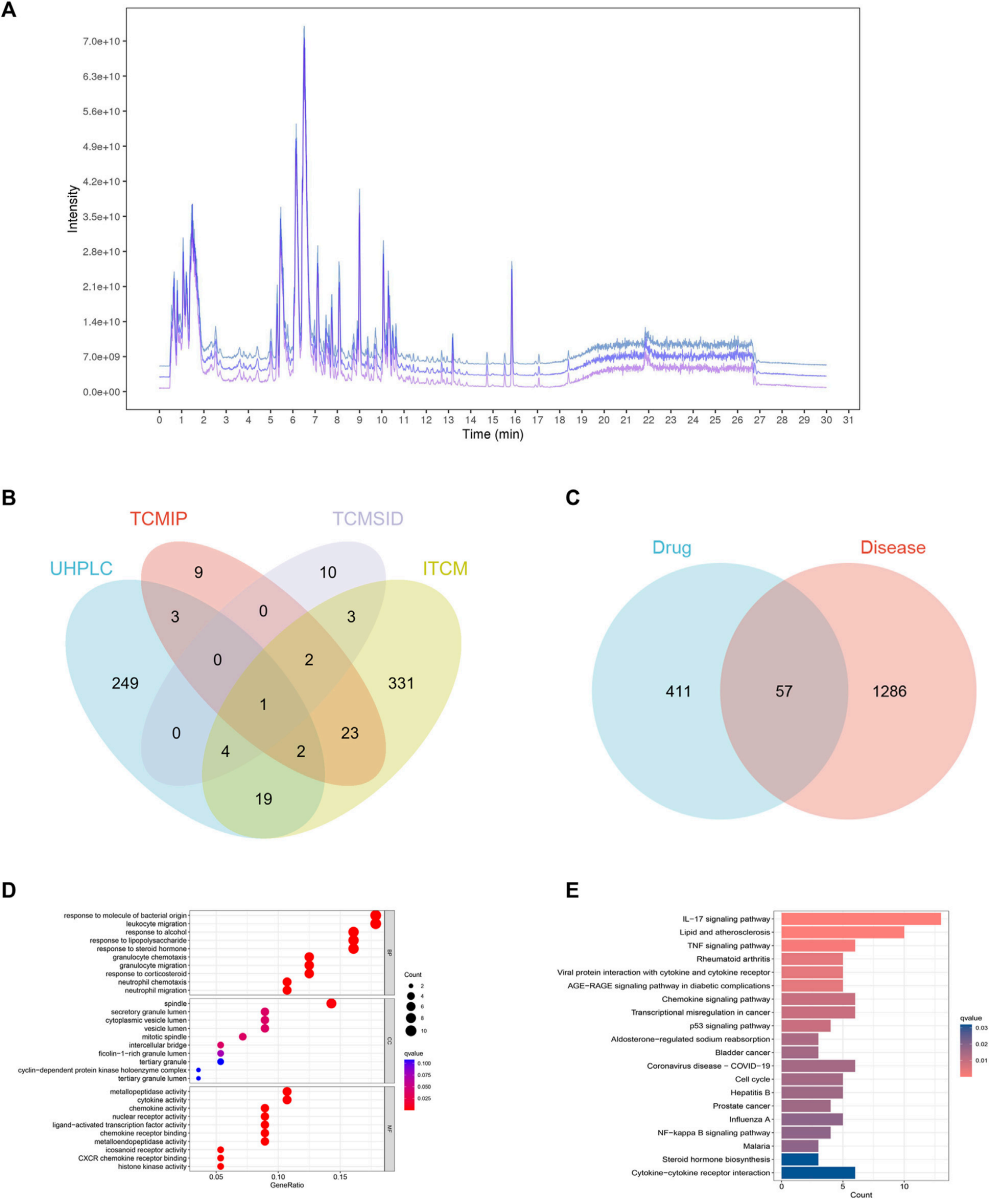
Network Analysis of FJDHF Therapy for Alzheimer’s Disease (AD): **(A)** TIC peaks of three batches of FJDHF analyzed by UHPLC-QE-MS; **(B)** Venn Maps of Compounds Collected from the ITCM, TCMIP, and TCMSID with Compounds Identified by Ultra High Performance Liquid Chromatography-Quadrupole-Exactive Orbitrap Mass Spectrometry (UHPLC-Q-Exactive Orbitrap MS); **(C)** Venn Diagram of FJDHF Targets and AD Disease Targets; **(D)** Gene Ontology (GO) and **(E)** Kyoto Encyclopedia of Genes and Genomes (KEGG) Enrichment Analyses were Performed for 57 Common Targets.

In this research, we utilized UHPLC-Q-Exactive Orbitrap MS to analyze three batches of FJDHF. Our analysis demonstrated the consistent presence of specific components across different batches (Figure 4A). To further investigate these ingredients, we compared them with three online databases (ITCM, TCMIP, and TCMSID). Through this comparison, we identified 29 duplicate ingredients that were selected for further research (Figure 4B; Supplementary Table S1).

A total of 407 candidate compounds were identified from three online databases: ITCM, TCMIP, and TCMSID. These were then intersected with compounds by UHPLC-Q-Exactive Orbitrap MS, and 29 compounds were selected for this study (Figure 4B; Supplementary Table S1). Subsequently, 468 potential targets were obtained by importing these compounds into the ITCM Database, the TargetNet Database, and the CTD online site (Figure 4C). The GSE32924 database was used to obtain AD-related genes, and the GSE32924 dataset was used to screen the differences in gene expression between the skin of AD patients in the skin lesions and the normal population, resulting in 655 upregulated and 688 downregulated genes (Supplementary Figure S1). A Venn map of disease differential genes and drug targets was created, with 57 common targets between the two. GO and KEGG enrichment analysis of these 57 genes showed that they mainly accumulated in IL-17 signaling pathways, responses to bacterial molecules, leukocyte migration, and other biological processes (Figures 4D, E).

### 3.4 Screening for hub genes in anti-AD action of FJDHF

**FIGURE 5:**
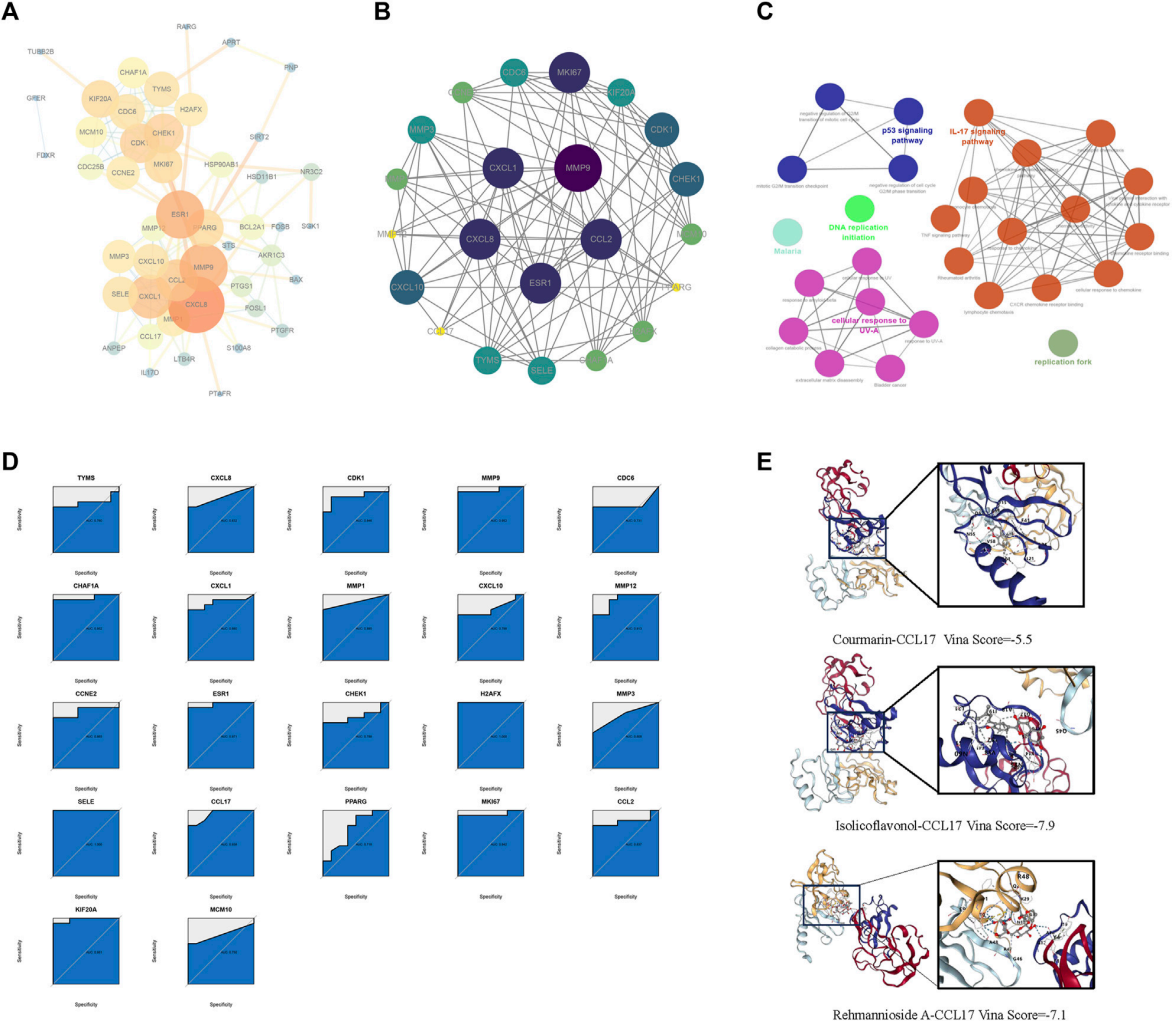
The target network for FJDHF on AD. **(A)** A PPI network of 57 targets was constructed through STRING. **(B)** MCODE was used to extract the most highly interconnected clusters form the network shown in A; **(C)** KEGG and GO enrichment analysis of 22 hub genes; **(D)** Diagnostic effects of each hub gene in the data set. **(E)** Molecular docking of FJDHF major key ingredients with CCL17. CCL17 is displayed as a color 3D model. The ingredient is depicted as a gray rod-shaped substance. Vina Score is the docking score.

To gain insight into how presumptive targets can mitigate AD, the 57 common targets obtained in 3.4 were used as input for the Search Tool for the STRING database to create a PPI network (Figure 5A). MCODE identified the most important clusters of highly interactive nodes, yielding 22 hub genes (Figure 5B). To explore their potential function, ClueGo was used to perform enrichment analyses, which showed that these genes are mainly associated with the IL-17 signaling pathway, p53 signaling pathway, and cellular response to UV-A (Figure 5C). The good clustering performance of these hub genes in the GSE28424 dataset was also confirmed by a ROC curve (Figure 5D; AUC >0.7). Then we performed molecular docking and confirmed that the key ingredients bind to CCL17 (Figure 5F).

### 3.5 Single cell sequencing analysis

**FIGURE 6:**
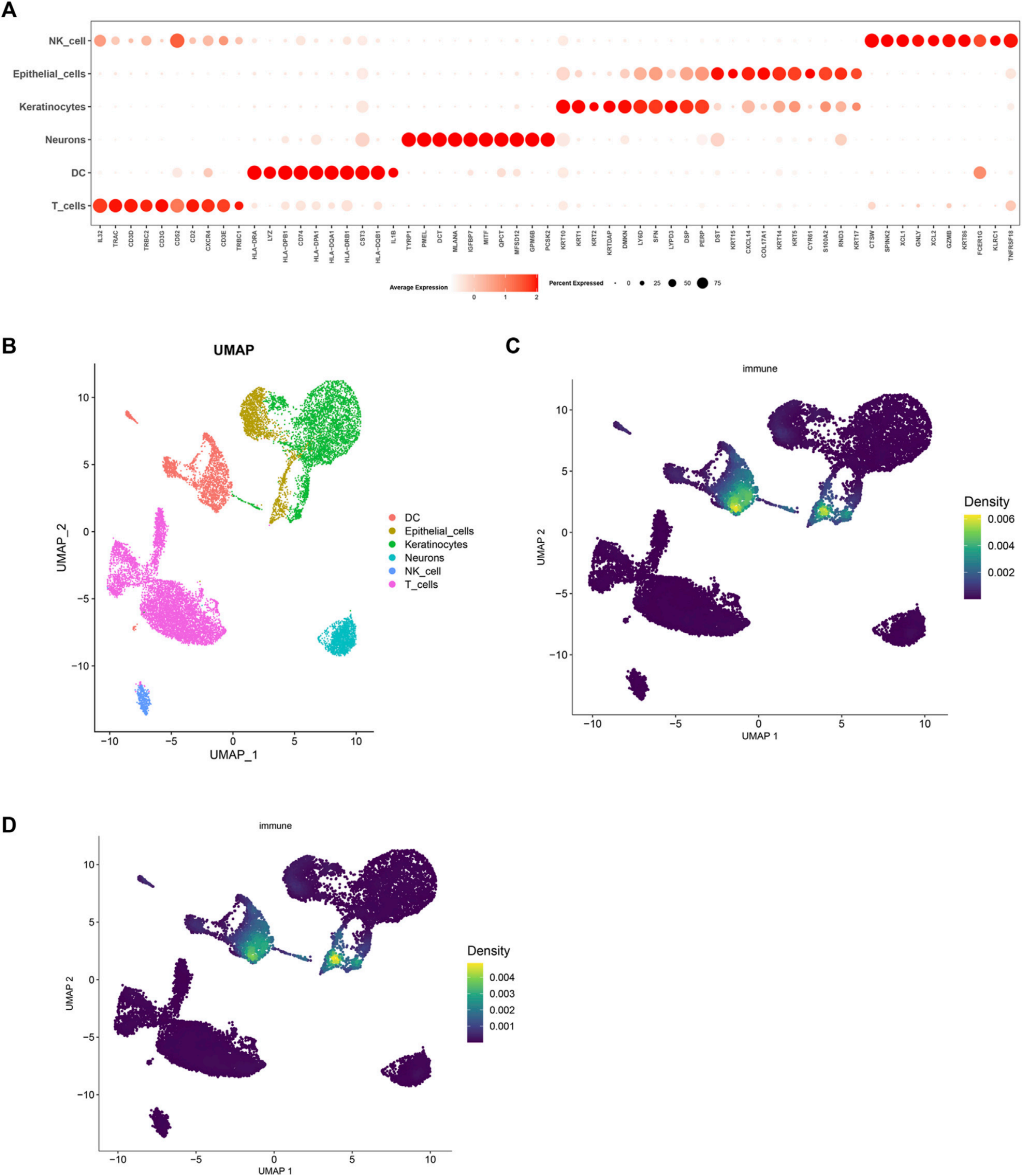
Analysis of single cell RNA sequencing in AD patients. **(A)** A bubble map illustrating marker genes expressed in different cells is presented. The intensity of the color indicates the average expression value of the marker gene, and the size of the circle corresponds to the percentage of cell expression of the marker gene; **(B)** Uniform manifold approximation and projection (UMAP) of single-cell RNA-seq data from AD patients, color code shows cell types; **(C)** AUCell and **(D)** UCell Scores.

**FIGURE 7:**
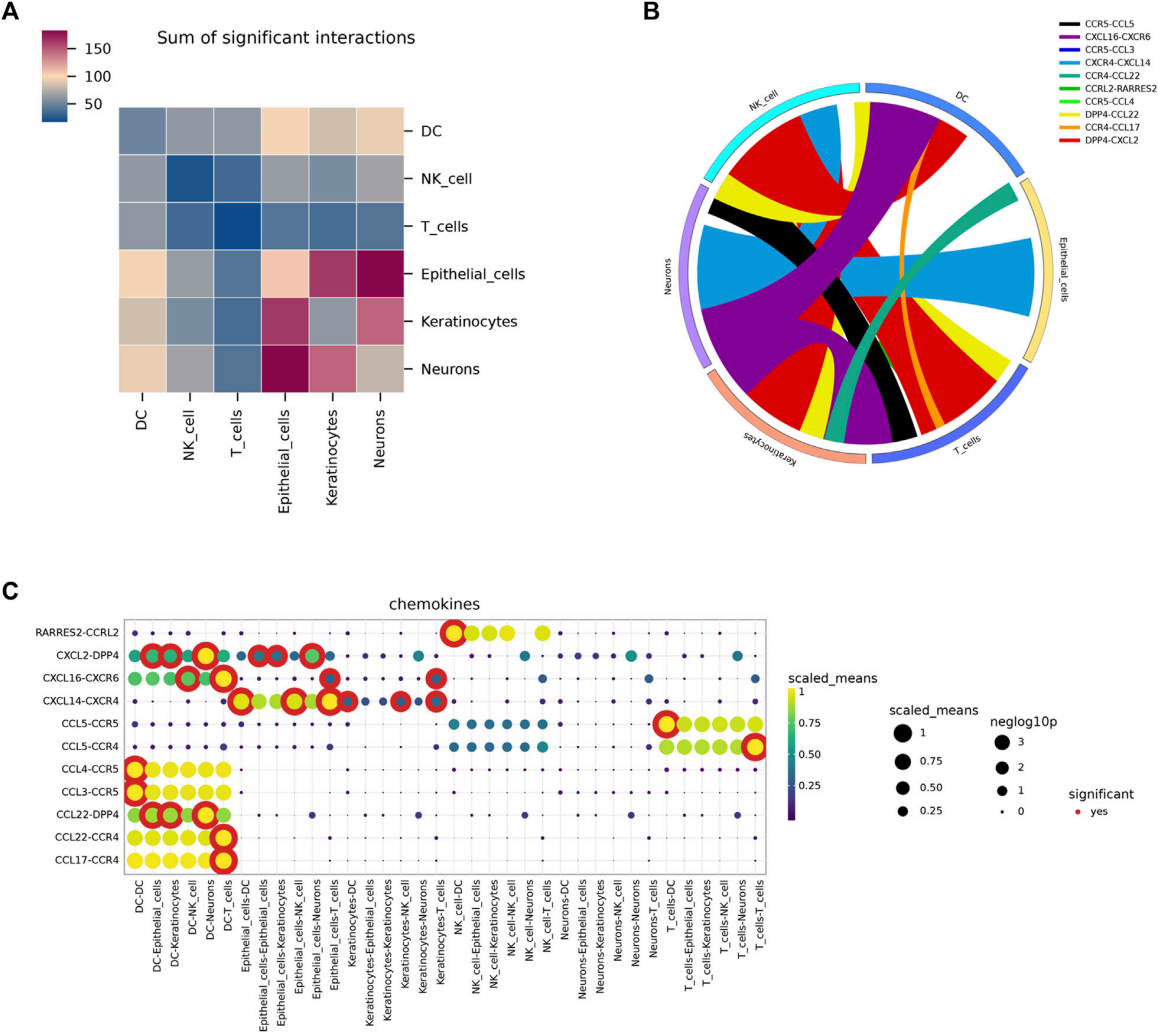
An overview of the interactions of various cell types analyzed using CellPhoneDB. **(A)** A heat map depicting cell interactions is used to illustrate the relationship between two different cell types. The two axes of the graph represent the two cell types, with the horizontal axis indicating the first type and the vertical axis indicating the second. The color gradient from dark blue to purple indicates the number of interactions, with dark blue indicating a low number and purple indicating a high number. **(B)** A circular diagram was used to represent the associations between receptor-ligand pairs between cells, with the color of the links representing the ligand object and the width of the links indicating the ligand-receptor interaction weight. **(C)** Dot plots illustrating putative ligand-receptor interactions between five cell types were generated, with the size of the dots representing the *p*-value and the color indicating the mean value of the receptor-ligand pairs between the two types of cells.

Following standardization and quality control, biopsy samples from the diseased area of 10 AD patients and skin tissues of healthy individuals were analyzed. Cluster analysis yielded six groups of cells, identified using Cellmaker annotation as dentritic cell, Epithelial_cells, Keratinocytes, Neurons, NK_cell and T_cells, with UMAP results obtained (Figures 6A, B). Cell distribution of drug target AUCell and UCell functional scores indicated that FJDHF mainly acted on DC, T cell and epithelial cell (Figures 6C, D). Moreover, the interaction between dendritic cells and T cells is accomplished by chemokines (Figures 7A–C). Coincidentally, we found that CCL17 is also one of our central genes, so we conducted molecular docking to verify the efficacy of our candidate compound and CCL17. The results showed that many compounds can effectively connect with CCL17 (Figure 7D).

### 3.6 FJDHF downregulates the proportion of Th1, Th2 and Th17 cells in AD model mice

**FIGURE 8:**
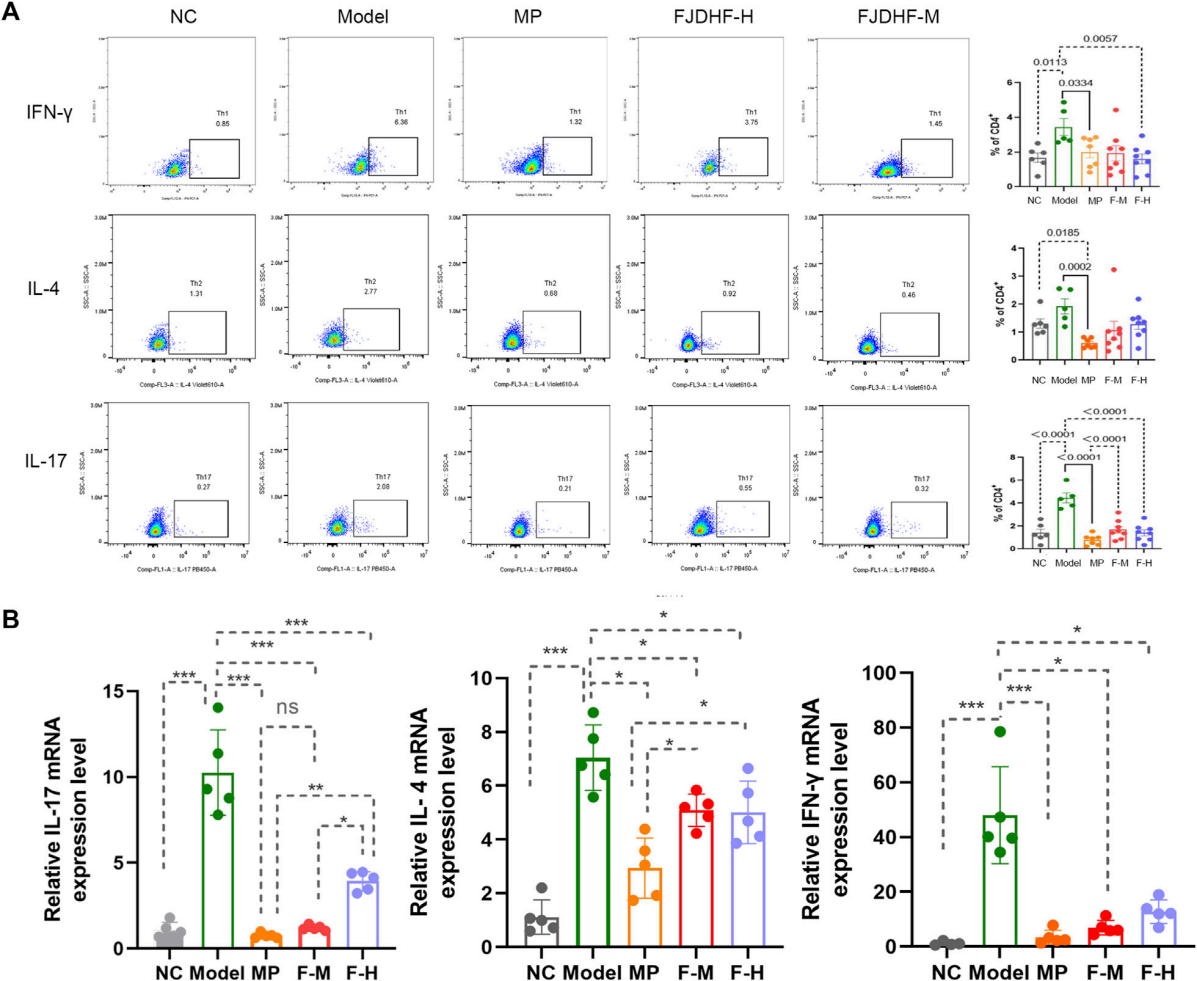
**(A)** The proportion of Th1, Th2 cells and Th17 cells in spleen of mice in each group. **(B)** The difference of IL-17, IL-4 and IFN-gamma mRNA expression in back skin of mice in each group.

On day 28 of the experiment, we performed sacrificial procedures on the mice and collected fresh spleens for flow analysis. The obtained results, as depicted in Figure 8, revealed significant increases in the proportions of Th1 (3.44% ± 0.72% vs. 1.70% ± 0.95%), Th2 (1.92% ± 1.23% vs. 1.05% ± 0.87%), and Th17 (4.27% ± 0.56% vs. 1.94% ± 1.05%) cells in the model group compared to the control group consisting of normal mice. Upon administering MP, FJDHF-H, and FJFHF-M, we observed a significant decrease in the ratio of Th1 and Th17 cells. Specifically, all three treatment groups exhibited comparable effects in reducing IFN-γ and IL-17 levels, with MP showing the most substantial decrease in Th17 cells, followed by FJDHF-M. FJFHF-H exhibited the weakest effect. These differences were statistically significant (n = 6-7, *p* < 0.05). Unfortunately, in terms of IL-4, although a reduction in data was observed, it did not reach statistical significance (Figure 8A).

We also examined the mRNA expression levels of IL-4 and IFN-γ in the back skin of mice in the four groups stimulated by DNCB and the NC group. The results showed that after the mice were exposed to DNCB, the expressions of IFN-γ and IL-4 in the back skin were significantly increased compared to the NC group, which confirmed the successful establishment of the AD model. The results of rt-PCR analysis confirmed that FJDHF treatment led to a reduction in the expression of IL-4 and IFN-γ in the back skin of the mice, compared to the model group (*p* < 0.05). Moreover, the expression level of IL-17 mRNA showed a significant decrease between model group and FJDHF-M (10.26 ± 2.48 vs. 1.20 ± 0.15, *p* < 0.05, Figure 8B). Therefore, we concluded that FJDHF effectively alleviated the impact of DNCB in the experimental mode.

### 3.7 FJDHF has an anti-atopic dermatitis (AD) role through the interleukin-17 (IL-17) signaling pathway

**FIGURE 9:**
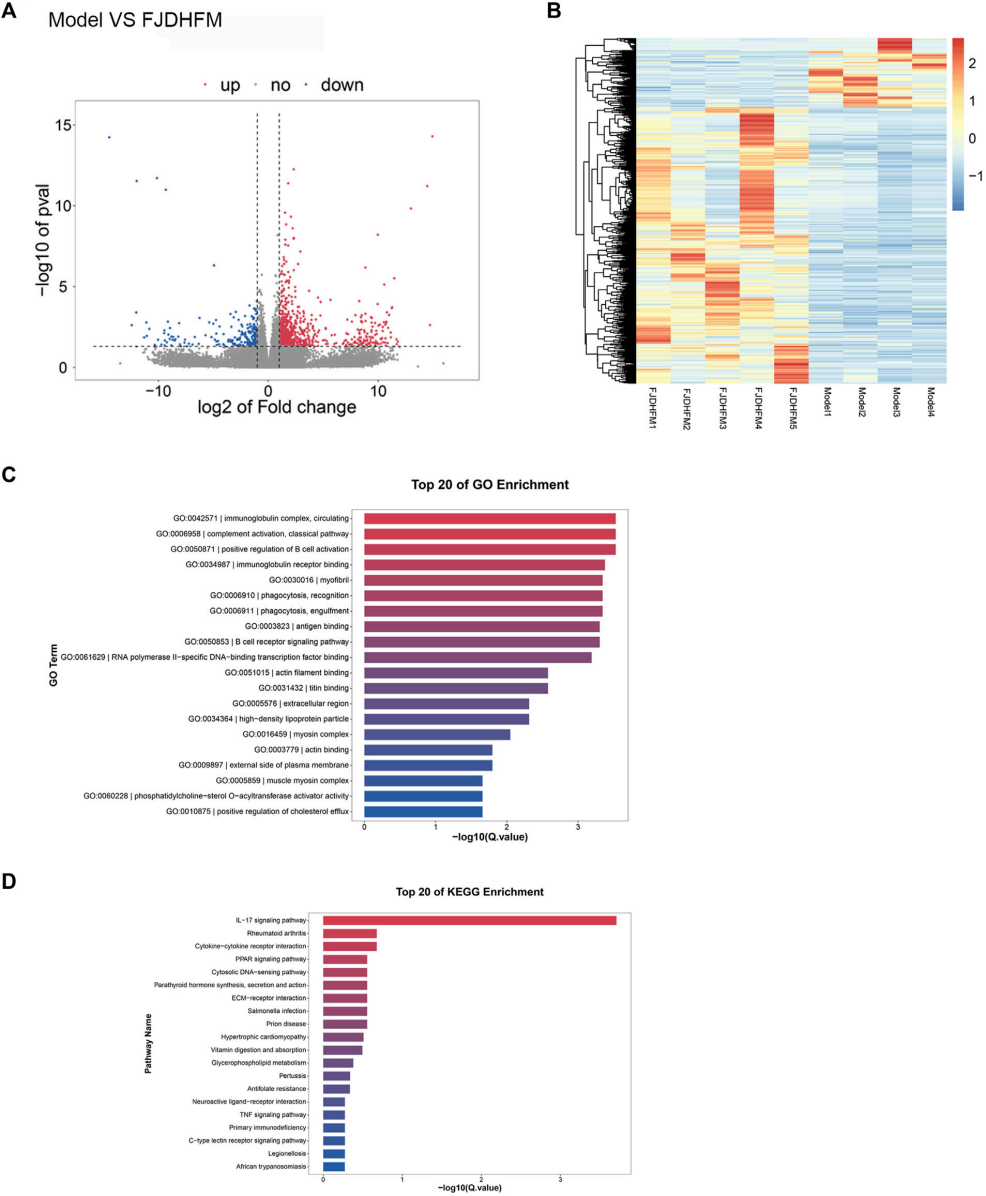
RNA sequencing of mouse back skin in model group and FJDHF-M group. Volcano map **(A)** and heat map **(B)** of DEGs in model group and FJDHF-M group; GO **(C)** and KEGG **(D)** enrichment analysis of DEGs.

The results of the *in vitro* experiment revealed that the efficacy of the FJDHF-M group was superior to that of the FJDHF-H group. To further investigate the potential underlying mechanisms, back skin of mice from the FJDHF-M group and the model group was collected for RNA-seq analysis. Our predictions were confirmed; significantly enriched DEGs in the FJDH group and the model group were found to be associated with the IL-17 pathway, cytokine receptor interactions, and the PPAR signaling pathway (Figure 9).

## 4 Discussion

Atopic dermatitis (AD) is a recurrent skin condition that is characterized by eczema lesions and intense pruritus (Ständer, 2021). The pathogenesis of AD is complex and involves genetic factors, epidermal dysfunction, skin microbiome abnormalities, and immune disorders. Currently, topical anti-inflammatory therapy is used as the first line of treatment for AD. Topical corticosteroids (TCS), topical calcineurin inhibitors tacrolimus and pimeclimus, and the more recently developed phosphodiesterase 4 (PDE4) inhibitor creborol are some of the treatments for AD (Bieber, 2022). For more severe forms of AD, current treatment guidelines recommend the use of cyclosporin A, methotrexate, azathioprine, mycophenolate, biologics, and ultraviolet light (Meng et al., 2021). Previous studies have shown that activated T helper cells in AD (mainly Th2, less Th1, Th17) trigger the release of interleukin and other inflammatory mediators, resulting in Th1/Th2 imbalance, which is an immunological hallmark of AD (Aranez and Ambrus, 2020). The use of biologics to target specific cytokines or Th1, Th2 or Th17 immune responses to suppress immune responses and reduce inflammation has been reported, but these agents can have a variety of side effects and rare complications (Bieber, 2022). Baritinib (JAK inhibitor) is an alternative approach that uses small molecules to interact with multiple signal transduction pathways linked to multiple cytokine receptors and immune pathways (Chun and Lehman, 2020). Although some patients may respond to drug therapy, immunomodulators and gastrointestinal side effects are common, which limits patients’ tolerance to oral preparations. In addition, more severe adverse events such as myosuppression, pulmonary fibrosis, and the risk of cancer and lymphoma have also been reported (Ratchataswan et al., 2021). As a result, the treatment of AD still needs further exploration.

Recently, FJDHF, a herbal prescription comprised of five botanical drugs, has been shown in pharmacological studies to inhibit inflammatory infiltration to varying degrees and reduce the levels of inflammatory cytokines such as TNF-α, IL-6, IL-1β and IFN-γ *in vivo* (Liu et al., 2020; Liu et al., 2020a; 2023; El-Saber Batiha et al., 2020; El-Saber Batiha et al., 2020; Liu et al., 2023). However, the mechanism of FJDHF is still not fully understood. Network pharmacology is a method of predicting underlying mechanisms based on analysis of the active ingredients in herbal prescriptions. Our study has improved the traditional approach of network pharmacology by firstly delineating the active constituents of FJDHF based on itcm and UHPLC-Q-Exactive Orbitrap MS. Secondly, the targets of FJDHF and those of AD were compared, and the therapeutic targets of FJDHF on AD were predicted. In order to identify the core of these targets, PPI maps were drawn for these targets and 22 hub genes were identified using the MCODE algorithm. Most of these 22 hub genes belong to cytokines, especially chemokines. Then, these hub genes were enriched and analyzed. The results showed that the drug could improve AD lesions by regulating IL-17 signaling pathway. A previous Chenglin Song study reported that FJDHF extract improves psoriasis related pathologic symptoms in a dose-dependent manner, possibly by inhibiting the IL-23/Th17 cell axis and reducing inflammatory cytokines for its effect (Song et al., 2021). Our results suggest that FJDHF plays a similar role in treating atopic dermatitis-like lesions as psoriasis. Previous studies have demonstrated that Th17 cells play a role in the pathogenesis of AD, particularly in pediatric and Asian populations (Liu et al., 2020). Furthermore, increased Th17 and decreased Th1 levels have been observed in the skin of Asian AD patients, which may explain the clinical efficacy of FJDHF in Chinese AD patients (Noda et al., 2015). Additionally, Britta C. Martel et al. observed that IL-22, IL-36a/g, IL-36RN and CCL22 expressions were similar between AD and psoriasis (Martel et al., 2016). Furthermore, our study demonstrated that FJDHF treatment effectively reduced the expression of IL-4 and IFN-γ in AD model mice. Previous research has highlighted the significance of IL-4 and IL-13, which serve as central cytokines in AD pathogenesis. These cytokines activate and stimulate Th2 cells, induce the differentiation and activation of myeloid and atopic dendritic cells, and recruit eosinophils. These immune responses subsequently contribute to skin infection, inflammation, skin thickening, and itching (Huang et al., 2022). Additionally, as the disease progresses into the chronic phase, the Th1 immune axis becomes dominant, leading to abnormal expression of IFN-γ (Bieber, 2008). These findings further confirm the substantial role of FJDHF in alleviating skin lesions in AD model mice.

Of note, bioinformatics analysis of single-cell RNA sequencing revealed that these targets were mainly active in dendritic cells. Studies of dendritic cells have revealed that they exhibit distinct immunomodulatory properties (Amon et al., 2019). Shruti Naik et al. demonstrated that exposure of dendritic cells loaded with S. epidermidis to CD8 T cells can result in the production of IL-17A and IFN-γ(Naik et al., 2015). Moreover, S. epidermidis is among the most common bacteria found on the skin of individuals with atopic dermatitis (AD). It has been demonstrated that S. epidermidis expresses high levels of cysteine protease activity upon growth at high cell densities. The enzyme responsible for this activity, EcpA, was identified and was found to be capable of degrading desmocoxin-1 and LL-37 *in vitro*, leading to the disruption of the epidermal barrier and inducing skin inflammation in mice (Cau et al., 2021). Furthermore, IL-23, predominantly secreted by DCs, is known to be involved in the modulation of many cellular processes and has been associated with various inflammatory skin disorders due to its capacity to bind to the IL-23R present on the surface of DCs (Anderson et al., 2018; Li et al., 2019). To investigate the relationship between DC cells and other skin tissues, we conducted a cell communication analysis. It is noteworthy that cytokines, particularly chemokines, are essential for the connection between DC cells and keratinocytes, epidermal cells, and T cells. Moreover, the anti-AD targets of FJDHF that we previously screened contained many chemokines. The potential of targeting the chemokine/chemokine receptor network for the treatment of atopic dermatitis is promising and of great developmental significance (Abboud and Hanson, 2017). Nevertheless, the crosstalk between DC, TL-17, and chemokines needs to be further explored in our subsequent experiments.

In conclusion, network pharmacology and bioinformatics analyses were used to elucidate the mechanism by which FJDHF downregulates IL-17 expression and ameliorates skin lesions in an animal model of atopic dermatitis. Our results provide evidence to support FJDHF as a potential therapeutic agent for atopic dermatitis.

## Data Availability

The data presented in the study are deposited in the GEO repository, accession number GSE244430.
