# Supplementary figures and images for: Fangji Dihuang formulation ameliorated DNCB-induced atopic dermatitis-like skin lesions by IL-17 signaling pathway: integrating network analysis and experimental validation
Source: Front Pharmacol. 2023 Nov 27;14:1220945. doi: 10.3389/fphar.2023.1220945 (PMC10711621; doi:10.3389/fphar.2023.1220945)

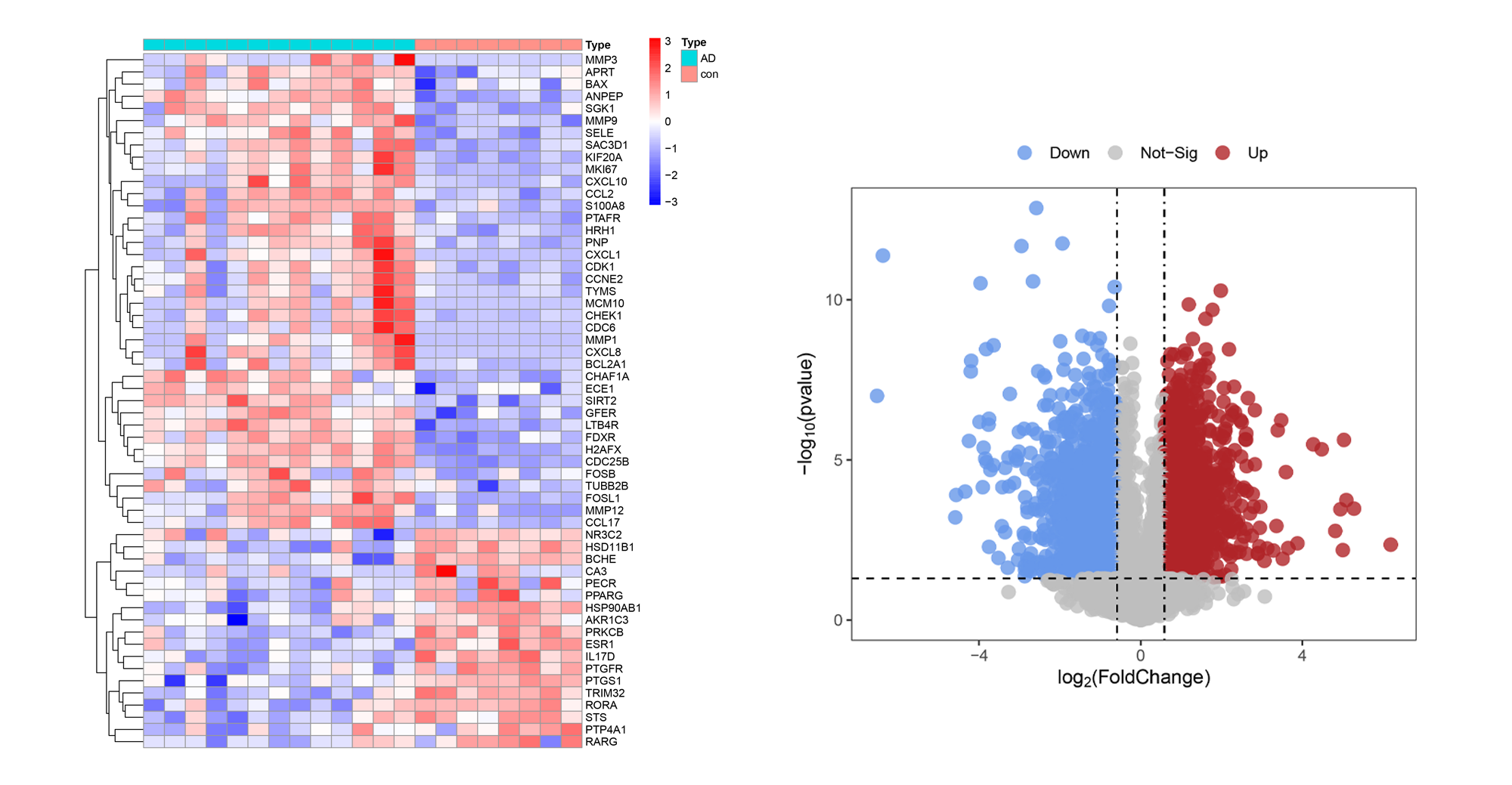

Supplement: Supplementary file 1 [file Image1.tif]
